# 2-[(1*H*-Benzimidazol-2-yl)imino­meth­yl]-4,6-dibromo­phenol ethanol hemisolvate

**DOI:** 10.1107/S1600536813002031

**Published:** 2013-02-09

**Authors:** Jie Liu, Zheng Liu, Shuai Yuan

**Affiliations:** a Key Laboratory of Nonferrous Metal Materials and Processing Technology, Department of Material and Chemical Engineering, Guilin University of Technology, Ministry of Education, Guilin 541004, People’s Republic of China

## Abstract

The title compound, C_14_H_9_Br_2_N_3_O·0.5C_2_H_5_OH, crystallizes with two 2-[(1*H*-benzimidazol-2-yl)imino­meth­yl]-4,6-dibromo­phenol mol­ecules and one ethanol solvent mol­ecule in the asymmetric unit. The benzene and benzimidazole rings subtend dihedral angles of 4.5 (3) and 5.2 (2)° in the two mol­ecules. In the crystal, one mol­ecule presents π–π stacking with the equivalent mol­ecule related by inversion, at a distance of 3.30 Å (separation between mol­ecular mean planes). A three-dimensional network is formed through N—H⋯N, N—H⋯O and O—H⋯N hydrogen bonds.

## Related literature

For background to benzimidazole compounds, see: Zhang *et al.* (2008 ▶, 2011 ▶).
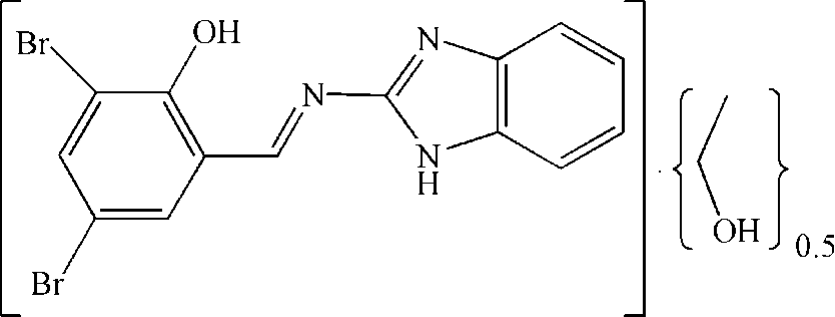


## Experimental

### 

#### Crystal data

C_14_H_9_Br_2_N_3_O·0.5C_2_H_6_O
*M* *_r_* = 836.19
Monoclinic, 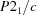

*a* = 14.5300 (15) Å
*b* = 16.8190 (14) Å
*c* = 13.8491 (17) Å
β = 111.656 (13)°
*V* = 3145.5 (6) Å^3^
*Z* = 4
Mo *K*α radiation
μ = 5.16 mm^−1^
*T* = 293 K
0.23 × 0.22 × 0.21 mm

#### Data collection

Agilent SuperNova Eos diffractometer
Absorption correction: multi-scan (*CrysAlis PRO*; Agilent, 2011 ▶) *T*_min_ = 0.436, *T*_max_ = 1.000
19465 measured reflections
6390 independent reflections
4189 reflections with *I* > 2σ(*I*)
*R*_int_ = 0.051

#### Refinement

*R*[*F* ^2^ > 2σ(*F* ^2^)] = 0.051
*wR*(*F* ^2^) = 0.133
*S* = 1.03
6390 reflections
392 parameters
H-atom parameters constrained
Δρ_max_ = 1.55 e Å^−3^
Δρ_min_ = −0.76 e Å^−3^

### 

Data collection: *CrysAlis PRO* (Agilent, 2011 ▶); cell refinement: *CrysAlis PRO*; data reduction: *CrysAlis PRO*; program(s) used to solve structure: *SHELXS97* (Sheldrick, 2008 ▶); program(s) used to refine structure: *SHELXL97* (Sheldrick, 2008 ▶); molecular graphics: *SHELXTL* (Sheldrick, 2008 ▶); software used to prepare material for publication: *SHELXTL*.

## Supplementary Material

Crystal structure: contains datablock(s) global, I. DOI: 10.1107/S1600536813002031/bh2461sup1.cif

Structure factors: contains datablock(s) I. DOI: 10.1107/S1600536813002031/bh2461Isup2.hkl

Supplementary material file. DOI: 10.1107/S1600536813002031/bh2461Isup3.cdx

Supplementary material file. DOI: 10.1107/S1600536813002031/bh2461Isup4.cml

Additional supplementary materials:  crystallographic information; 3D view; checkCIF report

## Figures and Tables

**Table 1:** Hydrogen-bond geometry (Å, °)

*D*—H⋯*A*	*D*—H	H⋯*A*	*D*⋯*A*	*D*—H⋯*A*
N5—H5⋯N3	0.86	2.14	2.997 (4)	172
N2—H2*A*⋯O3^i^	0.86	2.00	2.816 (4)	157
O1—H1⋯N1	0.82	1.90	2.618 (6)	146
O2—H2⋯N4	0.82	1.92	2.640 (6)	147
O3—H3⋯N6^ii^	0.82	2.08	2.900 (5)	176

Symmetry codes: (i) 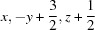; (ii) 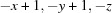.

**Fig. 1.**
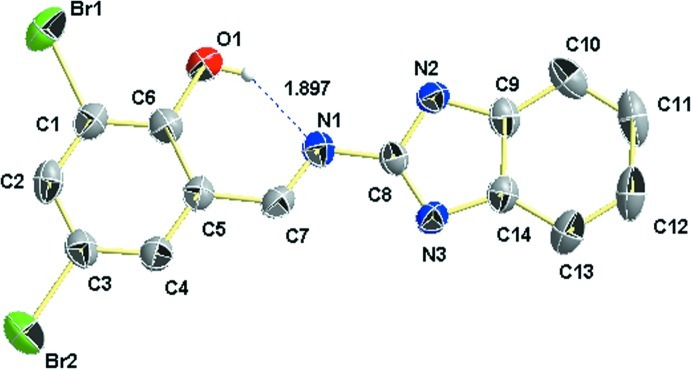
A view of one independent molecule in the title compound, showing 30% probability displacement ellipsoids.

**Fig. 2.**
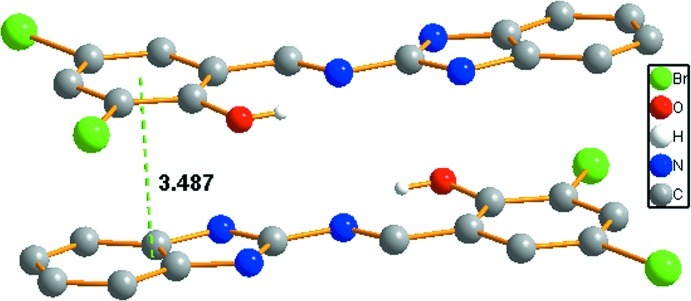
Perspective view of the stacking of two inversion-related molecules in the title compound.

**Fig. 3.**
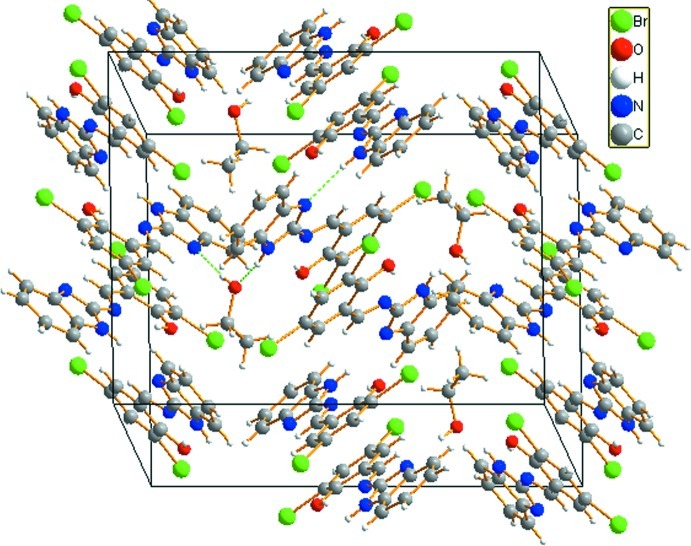
Three-dimensional network of the title compound, with dashed lines showing hydrogen bonds.

## supplementary crystallographic information

### Comment

Benzimidazole compounds exhibit a large range of biological activities and so far various types of benzimidazole drugs have been extensively used in clinic practice (Zhang *et al.*, 2008, 2011). Herein, we have synthesized a new benzimidazole compound.

In the title compound, the benzene and benzimidazole rings are almost in the same plane, and the dihedral angle between the benzene and benzimidazole rings is 4.5 (3) and 5.2 (2)° for each crystallographically independent molecule (Fig. 1). π–π stacking interactions are observed between the benzene and benzimidazole rings of molecules C1···C14 related by inversion: the dihedral angle between stacked molecules is 0°, and the separation between mean planes formed by the benzene and the benzimidazole rings in the molecules is 3.487 Å (Fig. 2). The title compound forms a three-dimensional network in the crystal structure, through N—H···N, N—H···O and O—H···N hydrgoen bonds (Fig. 3).

### Experimental

3,5-Dibromosalicylaldehyde (3.0 mmol) was dissolved in a round bottom flask containing ethanol (20 ml). To this solution, an ethanolic solution (10 ml) of 2-aminobenzimidazole (3.0 mmol) was then added dropwise, and the mixture was refluxed for 4 h. After cooling down to room temperature, the resulting orange precipitate was filtered off, and recrystallized from ethanol. Orange block-like crystals of the title compound were obtained by slow evaporation of the filtrate at room temperature.

### Refinement

H atoms on C, N, and O atoms were positioned geometrically and refined using a riding model (C—H*_aromatic_* = 0.93 Å, *C*—*H_ethanol_* = 0.97 Å, N—H = 0.86 Å and O—H = 0.82 Å) with *U*_iso_(H) = 1.2*U*_eq_(carrier atom), except for the methyl group in the ethanol molecule and hydroxyl groups, for which *U*_iso_(H) = 1.5*U*_eq_(carrier atom).

### Crystal data

**Table:**

C_14_H_9_Br_2_N_3_O·0.5C_2_H_6_O	*F*(000) = 1640
*M**_r_* = 836.19	*D*_x_ = 1.766 Mg m^−^^3^
Monoclinic, *P*2_1_/*c*	Mo *K*α radiation, λ = 0.71073 Å
Hall symbol: -P 2ybc	Cell parameters from 6390 reflections
*a* = 14.5300 (15) Å	θ = 2.8–29.1°
*b* = 16.8190 (14) Å	µ = 5.16 mm^−^^1^
*c* = 13.8491 (17) Å	*T* = 293 K
β = 111.656 (13)°	Block, orange
*V* = 3145.5 (6) Å^3^	0.23 × 0.22 × 0.21 mm
*Z* = 4	

### Data collection

**Table:**

Agilent SuperNova Eos diffractometer	6390 independent reflections
Radiation source: fine-focus sealed tube	4189 reflections with *I* > 2σ(*I*)
Graphite monochromator	*R*_int_ = 0.051
ω scans	θ_max_ = 26.4°, θ_min_ = 2.9°
Absorption correction: multi-scan (*CrysAlis PRO*; Agilent, 2011)	*h* = −17→18
*T*_min_ = 0.436, *T*_max_ = 1.000	*k* = −12→21
19465 measured reflections	*l* = −17→16

### Refinement

**Table:**

Refinement on *F*^2^	Primary atom site location: structure-invariant direct methods
Least-squares matrix: full	Secondary atom site location: difference Fourier map
*R*[*F*^2^ > 2σ(*F*^2^)] = 0.051	Hydrogen site location: inferred from neighbouring sites
*wR*(*F*^2^) = 0.133	H-atom parameters constrained
*S* = 1.03	*w* = 1/[σ^2^(*F*_o_^2^) + (0.0525*P*)^2^ + 1.2028*P*] where *P* = (*F*_o_^2^ + 2*F*_c_^2^)/3
6390 reflections	(Δ/σ)_max_ = 0.001
392 parameters	Δρ_max_ = 1.55 e Å^−^^3^
0 restraints	Δρ_min_ = −0.76 e Å^−^^3^
0 constraints	

### Fractional atomic coordinates and isotropic or equivalent isotropic displacement parameters (Å^2^)

**Table:**

	*x*	*y*	*z*	*U*_iso_*/*U*_eq_	
Br1	0.18346 (5)	0.58511 (4)	0.49389 (5)	0.0651 (2)	
Br2	0.16183 (5)	0.34709 (4)	0.19662 (5)	0.0773 (2)	
Br3	0.20356 (4)	0.66995 (4)	0.13312 (5)	0.0682 (2)	
Br4	0.02247 (4)	0.43016 (4)	−0.16402 (5)	0.0687 (2)	
O1	0.3836 (3)	0.6096 (2)	0.4833 (3)	0.0523 (9)	
H1	0.4378	0.6170	0.4793	0.078*	
O2	0.3869 (3)	0.5833 (2)	0.1429 (3)	0.0509 (9)	
H2	0.4369	0.5579	0.1491	0.076*	
O3	0.5513 (3)	0.7478 (2)	0.0641 (3)	0.0584 (10)	
H3	0.5114	0.7166	0.0249	0.088*	
N1	0.5251 (3)	0.5886 (2)	0.4114 (3)	0.0410 (10)	
N2	0.6683 (3)	0.6647 (2)	0.4789 (3)	0.0391 (9)	
H2A	0.6473	0.6908	0.5201	0.047*	
N3	0.6673 (3)	0.5779 (2)	0.3570 (3)	0.0383 (9)	
N4	0.4902 (3)	0.4627 (2)	0.1140 (3)	0.0380 (9)	
N5	0.6607 (3)	0.4473 (2)	0.2074 (3)	0.0398 (9)	
H5	0.6654	0.4877	0.2470	0.048*	
N6	0.5910 (3)	0.3573 (2)	0.0821 (3)	0.0365 (9)	
C1	0.2409 (3)	0.5300 (3)	0.4110 (4)	0.0430 (12)	
C2	0.1890 (3)	0.4700 (3)	0.3465 (4)	0.0463 (13)	
H2B	0.1258	0.4567	0.3430	0.056*	
C3	0.2322 (4)	0.4295 (3)	0.2863 (4)	0.0438 (12)	
C4	0.3270 (3)	0.4493 (3)	0.2931 (4)	0.0448 (12)	
H4	0.3557	0.4217	0.2533	0.054*	
C5	0.3790 (3)	0.5090 (3)	0.3576 (3)	0.0380 (11)	
C6	0.3357 (3)	0.5508 (3)	0.4174 (4)	0.0383 (11)	
C7	0.4786 (3)	0.5289 (3)	0.3606 (3)	0.0395 (11)	
H7	0.5078	0.4973	0.3248	0.047*	
C8	0.6193 (3)	0.6076 (3)	0.4121 (4)	0.0351 (10)	
C9	0.7585 (3)	0.6735 (3)	0.4691 (4)	0.0425 (12)	
C10	0.8406 (4)	0.7209 (3)	0.5210 (4)	0.0565 (14)	
H10	0.8415	0.7560	0.5732	0.068*	
C11	0.9220 (4)	0.7126 (4)	0.4902 (5)	0.0679 (18)	
H11	0.9792	0.7420	0.5233	0.081*	
C12	0.9177 (4)	0.6611 (4)	0.4110 (5)	0.0677 (18)	
H12	0.9721	0.6580	0.3912	0.081*	
C13	0.8372 (4)	0.6143 (3)	0.3604 (5)	0.0574 (15)	
H13	0.8368	0.5802	0.3074	0.069*	
C14	0.7557 (3)	0.6193 (3)	0.3904 (4)	0.0392 (11)	
C15	0.2131 (4)	0.5794 (3)	0.0568 (4)	0.0447 (13)	
C16	0.1299 (4)	0.5450 (3)	−0.0139 (4)	0.0472 (13)	
H16	0.0680	0.5671	−0.0260	0.057*	
C17	0.1377 (3)	0.4775 (3)	−0.0673 (4)	0.0444 (12)	
C18	0.2293 (3)	0.4427 (3)	−0.0496 (4)	0.0434 (12)	
H18	0.2339	0.3970	−0.0855	0.052*	
C19	0.3148 (3)	0.4774 (3)	0.0231 (3)	0.0367 (11)	
C20	0.3075 (3)	0.5466 (3)	0.0751 (4)	0.0387 (11)	
C21	0.4092 (3)	0.4375 (3)	0.0437 (4)	0.0410 (12)	
H21	0.4111	0.3926	0.0053	0.049*	
C22	0.5779 (3)	0.4202 (3)	0.1318 (4)	0.0351 (11)	
C23	0.6906 (3)	0.3406 (3)	0.1292 (3)	0.0341 (10)	
C24	0.7478 (4)	0.2809 (3)	0.1087 (4)	0.0473 (13)	
H24	0.7189	0.2432	0.0573	0.057*	
C25	0.8469 (4)	0.2791 (3)	0.1662 (4)	0.0517 (14)	
H25	0.8857	0.2395	0.1535	0.062*	
C26	0.8919 (4)	0.3357 (3)	0.2441 (4)	0.0478 (13)	
H26	0.9598	0.3326	0.2811	0.057*	
C27	0.8391 (3)	0.3952 (3)	0.2674 (4)	0.0440 (12)	
H27	0.8690	0.4325	0.3191	0.053*	
C28	0.7368 (3)	0.3966 (3)	0.2085 (3)	0.0357 (11)	
C29	0.5640 (5)	0.7298 (4)	0.1691 (5)	0.0677 (17)	
H29A	0.6296	0.7466	0.2143	0.081*	
H29B	0.5602	0.6726	0.1762	0.081*	
C30	0.4904 (6)	0.7678 (4)	0.2036 (5)	0.089 (2)	
H30A	0.4263	0.7453	0.1665	0.134*	
H30B	0.4888	0.8239	0.1900	0.134*	
H30C	0.5081	0.7592	0.2767	0.134*	

### Atomic displacement parameters (Å^2^)

**Table:**

	*U*^11^	*U*^22^	*U*^33^	*U*^12^	*U*^13^	*U*^23^
Br1	0.0689 (4)	0.0622 (4)	0.0823 (5)	0.0184 (3)	0.0492 (4)	0.0108 (3)
Br2	0.0635 (4)	0.0832 (5)	0.0757 (5)	−0.0212 (3)	0.0146 (4)	−0.0247 (4)
Br3	0.0597 (4)	0.0671 (4)	0.0849 (5)	0.0064 (3)	0.0350 (4)	−0.0178 (3)
Br4	0.0352 (3)	0.0942 (5)	0.0658 (4)	−0.0026 (3)	0.0059 (3)	−0.0097 (3)
O1	0.048 (2)	0.055 (2)	0.058 (2)	0.0017 (18)	0.025 (2)	−0.0100 (19)
O2	0.046 (2)	0.055 (2)	0.048 (2)	0.0020 (17)	0.0122 (19)	−0.0030 (18)
O3	0.062 (3)	0.062 (3)	0.047 (2)	−0.0218 (19)	0.016 (2)	−0.0002 (19)
N1	0.038 (2)	0.043 (3)	0.039 (2)	0.0001 (18)	0.011 (2)	0.0035 (19)
N2	0.039 (2)	0.041 (2)	0.038 (2)	0.0034 (18)	0.015 (2)	−0.0022 (18)
N3	0.036 (2)	0.041 (2)	0.040 (2)	0.0040 (17)	0.016 (2)	0.0000 (18)
N4	0.032 (2)	0.050 (3)	0.031 (2)	0.0020 (18)	0.0109 (19)	0.0026 (18)
N5	0.033 (2)	0.049 (3)	0.038 (2)	0.0006 (18)	0.0135 (19)	−0.0110 (19)
N6	0.028 (2)	0.044 (2)	0.036 (2)	−0.0030 (17)	0.0102 (18)	−0.0009 (18)
C1	0.039 (3)	0.052 (3)	0.038 (3)	0.011 (2)	0.014 (2)	0.011 (2)
C2	0.032 (3)	0.055 (3)	0.051 (3)	0.004 (2)	0.014 (3)	0.017 (3)
C3	0.037 (3)	0.053 (3)	0.034 (3)	−0.002 (2)	0.004 (2)	0.007 (2)
C4	0.038 (3)	0.062 (4)	0.033 (3)	0.000 (2)	0.012 (2)	0.002 (2)
C5	0.034 (3)	0.046 (3)	0.032 (3)	0.002 (2)	0.010 (2)	0.008 (2)
C6	0.039 (3)	0.037 (3)	0.034 (3)	0.005 (2)	0.007 (2)	0.010 (2)
C7	0.036 (3)	0.052 (3)	0.029 (3)	0.011 (2)	0.011 (2)	0.004 (2)
C8	0.029 (2)	0.037 (3)	0.035 (3)	0.001 (2)	0.007 (2)	0.003 (2)
C9	0.032 (3)	0.046 (3)	0.043 (3)	0.003 (2)	0.006 (2)	0.010 (2)
C10	0.052 (4)	0.051 (4)	0.051 (3)	−0.006 (3)	0.001 (3)	0.002 (3)
C11	0.037 (3)	0.071 (4)	0.081 (5)	−0.015 (3)	0.006 (3)	0.023 (4)
C12	0.033 (3)	0.077 (5)	0.092 (5)	0.002 (3)	0.022 (3)	0.020 (4)
C13	0.047 (3)	0.060 (4)	0.072 (4)	0.008 (3)	0.031 (3)	0.009 (3)
C14	0.033 (3)	0.041 (3)	0.041 (3)	0.007 (2)	0.010 (2)	0.008 (2)
C15	0.047 (3)	0.049 (3)	0.045 (3)	0.010 (2)	0.025 (3)	0.011 (2)
C16	0.033 (3)	0.062 (4)	0.048 (3)	0.010 (2)	0.017 (3)	0.009 (3)
C17	0.034 (3)	0.058 (3)	0.038 (3)	0.003 (2)	0.011 (2)	0.008 (2)
C18	0.039 (3)	0.049 (3)	0.043 (3)	0.002 (2)	0.016 (3)	0.003 (2)
C19	0.031 (3)	0.043 (3)	0.035 (3)	0.007 (2)	0.011 (2)	0.009 (2)
C20	0.031 (3)	0.049 (3)	0.033 (3)	−0.001 (2)	0.009 (2)	0.008 (2)
C21	0.040 (3)	0.049 (3)	0.036 (3)	0.003 (2)	0.016 (2)	−0.001 (2)
C22	0.030 (3)	0.042 (3)	0.033 (3)	−0.001 (2)	0.011 (2)	0.002 (2)
C23	0.031 (2)	0.039 (3)	0.030 (3)	−0.001 (2)	0.009 (2)	0.002 (2)
C24	0.051 (3)	0.041 (3)	0.051 (3)	0.004 (2)	0.020 (3)	−0.006 (2)
C25	0.044 (3)	0.051 (3)	0.064 (4)	0.017 (3)	0.025 (3)	0.007 (3)
C26	0.032 (3)	0.057 (4)	0.052 (3)	0.011 (2)	0.014 (3)	0.011 (3)
C27	0.032 (3)	0.059 (3)	0.039 (3)	0.000 (2)	0.010 (2)	−0.002 (2)
C28	0.029 (2)	0.047 (3)	0.033 (3)	0.006 (2)	0.013 (2)	0.000 (2)
C29	0.076 (4)	0.061 (4)	0.057 (4)	−0.012 (3)	0.013 (3)	0.008 (3)
C30	0.122 (6)	0.081 (5)	0.074 (5)	−0.020 (4)	0.047 (5)	−0.007 (4)

### Geometric parameters (Å, º)

**Table:**

Br1—C1	1.893 (5)	C9—C14	1.410 (7)
Br2—C3	1.891 (5)	C10—C11	1.405 (7)
Br3—C15	1.889 (5)	C10—H10	0.9300
Br4—C17	1.891 (5)	C11—C12	1.380 (8)
O1—C6	1.352 (6)	C11—H11	0.9300
O1—H1	0.8200	C12—C13	1.369 (8)
O2—C20	1.340 (5)	C12—H12	0.9300
O2—H2	0.8200	C13—C14	1.396 (6)
O3—C29	1.428 (6)	C13—H13	0.9300
O3—H3	0.8200	C15—C16	1.372 (7)
N1—C7	1.268 (6)	C15—C20	1.411 (6)
N1—C8	1.401 (5)	C16—C17	1.382 (7)
N2—C8	1.342 (6)	C16—H16	0.9300
N2—C9	1.374 (5)	C17—C18	1.389 (6)
N2—H2A	0.8600	C18—C19	1.406 (6)
N3—C8	1.309 (5)	C18—H18	0.9300
N3—C14	1.381 (6)	C19—C20	1.394 (7)
N4—C21	1.291 (6)	C19—C21	1.457 (6)
N4—C22	1.401 (5)	C21—H21	0.9300
N5—C22	1.350 (6)	C23—C24	1.397 (6)
N5—C28	1.392 (5)	C23—C28	1.414 (6)
N5—H5	0.8600	C24—C25	1.365 (7)
N6—C22	1.313 (6)	C24—H24	0.9300
N6—C23	1.380 (5)	C25—C26	1.406 (7)
C1—C2	1.373 (7)	C25—H25	0.9300
C1—C6	1.392 (6)	C26—C27	1.372 (7)
C2—C3	1.391 (7)	C26—H26	0.9300
C2—H2B	0.9300	C27—C28	1.407 (6)
C3—C4	1.386 (6)	C27—H27	0.9300
C4—C5	1.370 (7)	C29—C30	1.469 (8)
C4—H4	0.9300	C29—H29A	0.9700
C5—C6	1.401 (6)	C29—H29B	0.9700
C5—C7	1.470 (6)	C30—H30A	0.9600
C7—H7	0.9300	C30—H30B	0.9600
C9—C10	1.393 (7)	C30—H30C	0.9600
			
C6—O1—H1	109.5	C16—C15—C20	120.4 (5)
C20—O2—H2	109.5	C16—C15—Br3	120.8 (4)
C29—O3—H3	109.5	C20—C15—Br3	118.8 (4)
C7—N1—C8	120.3 (4)	C15—C16—C17	120.2 (4)
C8—N2—C9	107.0 (4)	C15—C16—H16	119.9
C8—N2—H2A	126.5	C17—C16—H16	119.9
C9—N2—H2A	126.5	C16—C17—C18	120.9 (5)
C8—N3—C14	104.0 (4)	C16—C17—Br4	119.9 (4)
C21—N4—C22	118.8 (4)	C18—C17—Br4	119.2 (4)
C22—N5—C28	106.2 (4)	C17—C18—C19	119.2 (5)
C22—N5—H5	126.9	C17—C18—H18	120.4
C28—N5—H5	126.9	C19—C18—H18	120.4
C22—N6—C23	104.3 (4)	C20—C19—C18	120.1 (4)
C2—C1—C6	121.3 (4)	C20—C19—C21	122.0 (4)
C2—C1—Br1	119.7 (4)	C18—C19—C21	117.9 (4)
C6—C1—Br1	119.1 (4)	O2—C20—C19	122.5 (4)
C1—C2—C3	119.2 (4)	O2—C20—C15	118.3 (5)
C1—C2—H2B	120.4	C19—C20—C15	119.2 (4)
C3—C2—H2B	120.4	N4—C21—C19	121.8 (4)
C4—C3—C2	119.9 (5)	N4—C21—H21	119.1
C4—C3—Br2	120.2 (4)	C19—C21—H21	119.1
C2—C3—Br2	119.9 (4)	N6—C22—N5	114.7 (4)
C5—C4—C3	121.0 (4)	N6—C22—N4	128.1 (4)
C5—C4—H4	119.5	N5—C22—N4	117.1 (4)
C3—C4—H4	119.5	N6—C23—C24	130.5 (4)
C4—C5—C6	119.5 (4)	N6—C23—C28	110.2 (4)
C4—C5—C7	119.1 (4)	C24—C23—C28	119.3 (4)
C6—C5—C7	121.4 (4)	C25—C24—C23	118.4 (5)
O1—C6—C1	118.5 (4)	C25—C24—H24	120.8
O1—C6—C5	122.4 (4)	C23—C24—H24	120.8
C1—C6—C5	119.1 (5)	C24—C25—C26	121.6 (5)
N1—C7—C5	121.0 (4)	C24—C25—H25	119.2
N1—C7—H7	119.5	C26—C25—H25	119.2
C5—C7—H7	119.5	C27—C26—C25	122.2 (5)
N3—C8—N2	114.4 (4)	C27—C26—H26	118.9
N3—C8—N1	129.7 (4)	C25—C26—H26	118.9
N2—C8—N1	115.9 (4)	C26—C27—C28	116.0 (5)
N2—C9—C10	132.6 (5)	C26—C27—H27	122.0
N2—C9—C14	104.5 (4)	C28—C27—H27	122.0
C10—C9—C14	122.9 (5)	N5—C28—C27	132.9 (4)
C9—C10—C11	116.3 (5)	N5—C28—C23	104.6 (4)
C9—C10—H10	121.9	C27—C28—C23	122.5 (4)
C11—C10—H10	121.9	O3—C29—C30	113.7 (5)
C12—C11—C10	120.5 (5)	O3—C29—H29A	108.8
C12—C11—H11	119.8	C30—C29—H29A	108.8
C10—C11—H11	119.8	O3—C29—H29B	108.8
C13—C12—C11	123.2 (5)	C30—C29—H29B	108.8
C13—C12—H12	118.4	H29A—C29—H29B	107.7
C11—C12—H12	118.4	C29—C30—H30A	109.5
C12—C13—C14	118.1 (6)	C29—C30—H30B	109.5
C12—C13—H13	120.9	H30A—C30—H30B	109.5
C14—C13—H13	120.9	C29—C30—H30C	109.5
N3—C14—C13	131.0 (5)	H30A—C30—H30C	109.5
N3—C14—C9	110.1 (4)	H30B—C30—H30C	109.5
C13—C14—C9	118.9 (5)		
			
C6—C1—C2—C3	−0.1 (7)	C20—C15—C16—C17	0.5 (7)
Br1—C1—C2—C3	−179.6 (3)	Br3—C15—C16—C17	−178.3 (4)
C1—C2—C3—C4	0.8 (7)	C15—C16—C17—C18	0.8 (7)
C1—C2—C3—Br2	−179.8 (4)	C15—C16—C17—Br4	179.6 (3)
C2—C3—C4—C5	−0.7 (7)	C16—C17—C18—C19	−0.5 (7)
Br2—C3—C4—C5	179.9 (4)	Br4—C17—C18—C19	−179.3 (3)
C3—C4—C5—C6	−0.2 (7)	C17—C18—C19—C20	−1.2 (7)
C3—C4—C5—C7	−178.8 (4)	C17—C18—C19—C21	177.1 (4)
C2—C1—C6—O1	−179.2 (4)	C18—C19—C20—O2	−178.1 (4)
Br1—C1—C6—O1	0.3 (6)	C21—C19—C20—O2	3.8 (7)
C2—C1—C6—C5	−0.7 (7)	C18—C19—C20—C15	2.5 (7)
Br1—C1—C6—C5	178.8 (3)	C21—C19—C20—C15	−175.7 (4)
C4—C5—C6—O1	179.2 (4)	C16—C15—C20—O2	178.3 (4)
C7—C5—C6—O1	−2.2 (7)	Br3—C15—C20—O2	−2.8 (6)
C4—C5—C6—C1	0.9 (7)	C16—C15—C20—C19	−2.2 (7)
C7—C5—C6—C1	179.4 (4)	Br3—C15—C20—C19	176.7 (3)
C8—N1—C7—C5	−178.9 (4)	C22—N4—C21—C19	178.2 (4)
C4—C5—C7—N1	173.0 (5)	C20—C19—C21—N4	2.6 (7)
C6—C5—C7—N1	−5.5 (7)	C18—C19—C21—N4	−175.6 (4)
C14—N3—C8—N2	−0.1 (5)	C23—N6—C22—N5	0.6 (5)
C14—N3—C8—N1	179.9 (5)	C23—N6—C22—N4	178.9 (4)
C9—N2—C8—N3	−1.0 (6)	C28—N5—C22—N6	−0.4 (5)
C9—N2—C8—N1	179.0 (4)	C28—N5—C22—N4	−178.9 (4)
C7—N1—C8—N3	8.9 (8)	C21—N4—C22—N6	3.2 (7)
C7—N1—C8—N2	−171.1 (4)	C21—N4—C22—N5	−178.5 (4)
C8—N2—C9—C10	−177.0 (5)	C22—N6—C23—C24	−179.3 (5)
C8—N2—C9—C14	1.6 (5)	C22—N6—C23—C28	−0.5 (5)
N2—C9—C10—C11	178.9 (5)	N6—C23—C24—C25	178.3 (5)
C14—C9—C10—C11	0.5 (7)	C28—C23—C24—C25	−0.4 (7)
C9—C10—C11—C12	1.4 (8)	C23—C24—C25—C26	−0.2 (8)
C10—C11—C12—C13	−1.7 (9)	C24—C25—C26—C27	0.5 (8)
C11—C12—C13—C14	0.1 (9)	C25—C26—C27—C28	−0.2 (7)
C8—N3—C14—C13	−179.8 (5)	C22—N5—C28—C27	178.2 (5)
C8—N3—C14—C9	1.2 (5)	C22—N5—C28—C23	0.0 (5)
C12—C13—C14—N3	−177.1 (5)	C26—C27—C28—N5	−178.5 (5)
C12—C13—C14—C9	1.8 (7)	C26—C27—C28—C23	−0.5 (7)
N2—C9—C14—N3	−1.8 (5)	N6—C23—C28—N5	0.3 (5)
C10—C9—C14—N3	177.0 (4)	C24—C23—C28—N5	179.3 (4)
N2—C9—C14—C13	179.1 (4)	N6—C23—C28—C27	−178.1 (4)
C10—C9—C14—C13	−2.1 (7)	C24—C23—C28—C27	0.8 (7)

### Hydrogen-bond geometry (Å, º)

**Table:**

*D*—H···*A*	*D*—H	H···*A*	*D*···*A*	*D*—H···*A*
N5—H5···N3	0.86	2.14	2.997 (4)	172
N2—H2*A*···O3^i^	0.86	2.00	2.816 (4)	157
O1—H1···N1	0.82	1.90	2.618 (6)	146
O2—H2···N4	0.82	1.92	2.640 (6)	147
O3—H3···N6^ii^	0.82	2.08	2.900 (5)	176

Symmetry codes: (i) *x*, −*y*+3/2, *z*+1/2; (ii) −*x*+1, −*y*+1, −*z*.
